# Trust in scientific institutions and experts during the first 2 years of the COVID-19 pandemic in Finland

**DOI:** 10.1177/14034948241289633

**Published:** 2024-10-24

**Authors:** Piia T. Jallinoja, Eetu V. Vento, Esa T. Väliverronen

**Affiliations:** 1 Tampere University, Finland; 2 University of Helsinki, Finland

**Keywords:** Trust in science, COVID-19, experts, political affiliation, sociodemographic factors, health policy, vaccinations, health authorities

## Abstract

**Aims:**

To explore variations in trust in science and scientific institutions in Finland during the COVID-19 pandemic, and the factors influencing trust in experts of a key institution in the management of the pandemic, the Finnish Institute for Health and Welfare (THL). These results are contrasted with trust in the Ministry of Social Affairs and Health.

**Methods:**

Five surveys were conducted between April 2020 and March 2022 (*n*=5448). The changes were tested with the chi-square test. Predictors of trust in THL and the ministry was examined with binary logistic regression.

**Results:**

Trust in science and key scientific institutions remained consistently high throughout this period. In the early pandemic, trust in the ministry declined. The most significant explanatory factors for trust in THL and the ministry were being a supporter of some other party than the right-wing Finns Party and belonging to the age groups over 50 years.

**Conclusions:**

**Our findings indicate that the pandemic, marked by unpredictability, did not weaken trust in science and THL, whereas trust in the ministry responsible for managing the pandemic and university experts weakened. In increasingly politically polarised societies, the impact of political sentiments on health-related perceptions and choices should be analysed more in future public health studies.**

## Introduction

Scientific institutions and their experts have emerged as key players in the fight against the COVID-19 pandemic. However, concerns have been raised about the potential erosion of public trust in science during this period. This erosion can lead to non-adherence in vaccination programmes and other guidelines to reduce transmission. This paper examines the variation of trust in science and scientific institutions, specifically those with a central role in managing the pandemic, between April 2020 and March 2022.

During the pandemic, the Finnish Institute of Health and Welfare (THL) and universities were the primary scientific authorities in public health. THL played a crucial role as a research and development institute operating under the Ministry of Social Affairs and Health (the ministry), providing data and predictions. Besides this central role, in the early stages of the pandemic, THL and the ministry were criticised for underestimating the severity of the pandemic and responding too slowly. Critics, including the ‘End COVID-19 Finland’ group of professors, researchers and medical doctors advocated for stronger measures, causing some consensus differences. In Autumn 2021, disagreements between THL and the ministry over pandemic restrictions exacerbated a similar situation. The ministry was advocating for a more stringent response to the pandemic, while THL was proposing a more relaxed approach [1].

In addition, various citizen groups criticised the pandemic policies for being too lenient or too strict. Some spread conspiracy theories, or criticised the corrupt mainstream media, the suppression of freedom of speech and the influence of the pharmaceutical industry in the vaccination programme. The US-based QAnon activism and Canada-originated Freedom Convoy March inspired some of the voices.

In this paper, we define trust as an individual’s mental disposition towards the trustee, the object of trust. This disposition is rooted in positive expectations of the trustee, particularly under conditions of risk and uncertainty [2]. Importantly, trust is a multifaceted phenomenon: the trustors evaluate the trustee’s competence, reliability, performance and commitment to an obligation [3].

In Finland the level of trust in science and expert institutions was high before the COVID-19 pandemic [4, 5]. The pandemic period can be postulated to have either diminished or strengthened public trust in science.

Indicators of a potential decline include the spread of misinformation and a variety of conspiracy theories during the pandemic [6], which could result in a decrease in trust. Furthermore, the pandemic incited political populism [7] and political polarisation [8], potentially fostering scepticism towards science and scientific authorities [9]. Other factors potentially contributing to an erosion of trust in science include scientific uncertainty caused by the pandemic [10], disagreement among experts [11] and constant changes in policy recommendations [10]. In addition, mental exhaustion of the circumstances, ‘pandemic fatigue’, has been linked to anti-establishment attitudes [12].

Researchers’ and scientific organisations’ efforts to protect public health and their positive visibility in the media could also contribute to strengthening trust in science [10]. A similar impact could be attributed to the ‘rally around the flag’ phenomenon, which refers to citizens uniting in support of their authorities during significant crises [13]. In addition, in many European countries, the lack of polarisation by populist parties in relation to the pandemic could potentially safeguard against a decline in trust in science and experts [14].

Earlier studies on the topic have shown relatively mixed results. Most studies have reported that trust either decreased or remained relatively stable [15–18], while others showed the initial rise in trust [19]. Some reported an initial rise followed by weakening as the pandemic progressed [20, 21]. These somewhat divergent results may be explained by differences in the pre-pandemic level of trust in science, pandemic policies and different ways of conceptualising and measuring trust in questionnaires.

Trust in science varies among different population groups. In the US, COVID-19 scepticism correlated with political conservatism [22]. In Finland, during the early pandemic, the supporters of the right-wing populist party were more prone to have sceptical opinions of public health authorities regarding the COVID-19 issues [23]. Furthermore, young age [22, 24], low scientific knowledge [24] and a low level of education [24] have been shown to be associated with COVID-19 scepticism.

This article first asks how trust in science, scientific institutions and experts varied from April 2020 to March 2022. In addition, the objective is to examine trust towards executive government, the Ministry of Social Affairs and Health. Second, the objective is to explore how the sociodemographic and political party preference predict trust in a scientific institution, THL and the ministry.

By analysing trust in scientific institutions in diverse ways, and in the executing government at five time points during the first 2 years of the pandemic, and including political sentiments in the analysis, this study provides a unique perspective on public health studies related to the COVID-19 pandemic.

### The Finnish context of the COVID-19 pandemic

**Figure 1.**
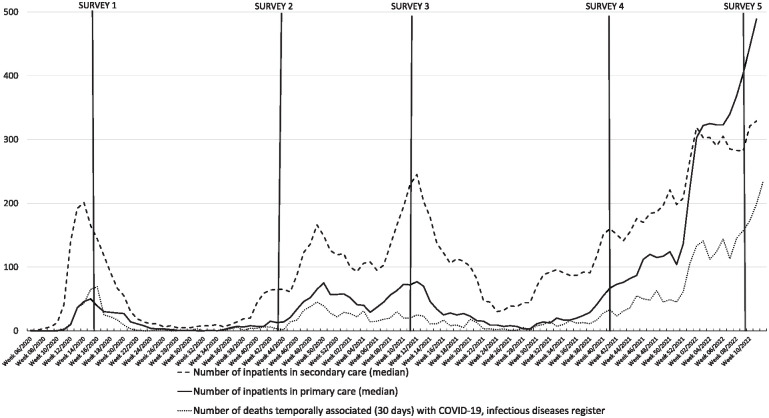
Number of inpatients in primary and secondary care, and deaths temporarily associated (30 days) with COVID-19 from week 6 2020 to week 45 2022 and the timing of the surveys [27].

The COVID-19 epidemic started in Finland in mid-March 2020 (Table I, Figure 1). By European comparison, Finland ‘survived’ the pandemic relatively well, with 5050 deaths attributed primarily to COVID-19 by the end of 2022 (in a population of 5.6 million) [25].

**Table I.** The progression of the pandemic in Finland and the scheduling of the surveys [1, 25–27].

January–April 2020	The pandemic began, and the government declared a state of emergency on 16 March. Restrictions were imposed, such as the prohibition of public gatherings of more than 10 people, closure of restaurants and educational institutions, and restrictions on movement to and from Uusimaa (the county around the capital district), as well as over the national borders. The national mood and the media coverage of the pandemic was in crisis mode.
**The first survey, 9–14 April 2020, *n*=1138** The first peak in the number of inpatients in primary and secondary care had just been passed	
April–August 2020	Many of the restrictions were lifted during late spring and early summer. In summer 2020, despite the low number of deaths and hospitalisations, the pandemic received extensive media coverage.
August–October 2020	The second wave commenced. The opposition and some other groups criticised the government’s response to the pandemic, such as its reluctance to recommend the use of facial masks. In the end, the mask recommendations gradually became tighter.
**The second survey, 23–28 October 2020, *n*=1052** The early phase of the second peak in the number of inpatients in primary and secondary care	
October–December 2020	In late 2020, the situation worsened with COVID-19-related deaths and hospitalisations reaching their peak in December.
January–March 2021	In January 2021, the vaccination programme began. However, in March 2021, THL suspended vaccinations with AstraZeneca’s products as a precautionary measure following reports of blood clotting in the brain associated with the vaccine. Numbers of hospitalised patients rose, reaching a new peak in late March. On 1 March 2021, a new state of emergency was announced. Some protective measures and restrictions on movement were introduced, including the closure of restaurants.
**The third survey, 19–25 March 2021, *n*=1151** The height of the third peak in the number of inpatients in primary and secondary care. Less than 10% of the population vaccinated.	
April–July 2021	In April, the use of AstraZeneca vaccine was resumed, but only for people aged 65 years or over. The restrictions were lifted during the spring as the overall situation began to improve slowly.
August–October 2021	The arrival of the Delta variant led to a worsening of the pandemic during the late summer and early autumn of 2021. The first signs of pandemic fatigue and waning interest in the pandemic were observed. The vaccination programme was well underway.
**The fourth survey, 15–19 October 2021, *n*=1044** The fourth peak had begun with inpatients in primary and secondary care rising. 75% of the population vaccinated once, 64% vaccinated twice.	
October–December 2021	Growing disagreements arose concerning the pandemic restrictions between the Ministry of Social Affairs and Health and THL, with the ministry pushing for a stricter pandemic response while THL argued for a more lenient approach. In October, the government lifted pandemic restrictions, only to reinstate them in late November.
January–March 2022	In late 2021 and early 2022, the Delta and Omicron variants swept through Finland, causing the number of COVID-19-related deaths and hospitalisations to increase steeply. Despite COVID-19-related deaths and hospitalisations being at an all-time high during spring 2022, most remaining restrictions were lifted.
**The fifth survey, 4–9 March 2022, *n*=1063** The fourth peak continues. 82% of the population vaccinated once, 78% vaccinated twice.	

Regarding the political context of the period, a five-party centre-left government led by Prime Minister Sanna Marin (the Social Democratic Party) governed Finland [26]. Other parties in the government were the Centre Party, the Green League, the Left Alliance and the liberal Swedish People’s Party.

Initially, the opposition parties, including the centre-right National Coalition Party and the right-wing populist Finns Party, refrained from criticising the government’s actions, adhering to an ethos of ‘combating the devil together’. Later, the opposition criticised the government’s pandemic policies, such as the continuation of restrictions. However, none of the major political parties positioned themselves against vaccinations.

## Data and methods

Five surveys were conducted: 9–14 April 2020 (n=1138); 23–28 October 2020 (*n*=1052); 19–25 March 2021 (*n*=1151); 15–19 October 2021 (*n*=1044); and 4–9 March 2022 (*n*=1063), by Kantar Oy (now Verian) among the members of its consumer panel living in mainland Finland. The survey aimed for a representative sample of age, gender and geographical area. Some respondents may have answered several surveys, but due to anonymity we cannot verify this. In each survey, panel members were sent one reminder message.

The panel members are asked annually about background factors and voting intentions. As the respondents were relatively similar in their background factors in each survey, the information is combined (Table II). A comparison with Statistics Finland’s reports [28] showed that the survey data are satisfactorily representative in terms of gender, age and geographical area. It was somewhat challenging to compare the respondents’ education level with Statistics Finland reports [28], but it seems that those who had only an elementary level of education were underrepresented in the survey, while those with an academic degree were overrepresented. Comparison of voting intentions with the results of the municipal elections of 2021 proved difficult because of the options ‘I can’t say’, ‘I don’t intend to vote’ and ‘I don’t want to say’ in the survey. Nevertheless, the parties’ popularity order in our data resembles satisfactorily that of the 2021 elections.

**Table II.** Background characteristics and voting intentions of the respondents, all surveys, percentages, *n*=5448.

	Surveys 1–5together	Population dataStatistics Finland 31.12.2021

Gender		≥20 years old
Women	48	51
Men	52	49
Age		The youngest age group is 20–29 years
Under 30 years	14.2	14.8
30–39 years	15.2	16.6
40–49 years	14.6	15.4
50–59 years	17.3	16.1
60–69 years	18.8	16.1
70 years or older	19.9	21
Geographical area		≥20 years old
Uusimaa region, including capital area	34.7	32.4
Southern Finland (other than Uusimaa)	19.6	20.5
Western Finland	23.0	24.6
Eastern and Northern Finland	22.7	22.5
Education		≥15 years old
Elementary school	9	21
Vocational school	19.6	40.5 (vocational school and
Matriculation examination	11.1	matriculation exam together)
Post-secondary vocational school	16.5	14.3
University of applied sciences, Barchelor's degree	22	12.5
University (Master's degree or higher)	21.2	11.7
Other	0.6	
Voting intentions		Municipal elections 2021
The Finns Party	11.4	14.5
The National Coalition Party	14.6	21.4
The Centre Party	7.5	14.9
The Green League	9.9	10.6
The Social Democratic Party	12.4	17.7
The Left Alliance	6.1	7.9
Other parties	6.6	13.0
Would not vote now	3.3	n.a.
Don’t know	13.4	n.a.
Don’t want to say	14.7	n.a.

The questionnaire included questions regarding perceptions of public authorities, experts, science, medicine, media, COVID-19 measures, the nature of the pandemic and beliefs typical of conspiratorial thinking – mostly in the context of COVID-19. Each survey had 10 sections, each containing five to 10 statements. To avoid lengthiness, some old questions were omitted as new ones were introduced. Some previous questions on science and science institutions were applied [5]. The questionnaire was not pre-tested due to the need to capture early pandemic stages quickly, but each item’s validity was reviewed before each survey.

For this study seven items measuring trust in science, science institutions and experts were included. These included four statements suggesting trust: ‘The most reliable experts in predicting and managing the coronavirus pandemic are university professors and researchers’; ‘The most reliable experts in predicting and managing the coronavirus pandemic are the representatives of THL’; ‘I trust the information provided by the authorities and experts about vaccines and vaccinations [in the COVID-19 context]’; and ‘Science and research in our country are characterised by efficiency and a high level of professional expertise’; and three statements indicating distrust: ‘Science cannot be trusted because experts in the same field may disagree completely on something [in the COVID-19 context]’; ‘A group of researchers is deliberately trying to create panic and concern about the coronavirus pandemic’; and ‘An expert does not necessarily need to have a scientific education, as practical experience and insight are more important [in the COVID-19 context]’. The answering options were: ‘completely disagree’; ‘somewhat disagree’; ‘somewhat agree’; ‘completely agree’ and ‘I don’t know’ (‘completely agree’ and ‘somewhat agree’ were combined for the analysis). Opinions on THL and the ministry were measured with a question ‘How well do you think the following parties have succeeded in handling the corona crisis’ followed by a list of institutions. The answering options were: ‘I don’t know’; ‘very poorly’; ‘rather poorly’; ‘neither well, not poorly’; ‘rather well’ and ‘very well’. In the analysis the last two options are combined.

We included the following background variables in the regression analyses: gender, age (classified as in Table I) and education, reclassified as basic level (elementary school, vocational school matriculation examination), middle level (postsecondary vocational school, university of applied sciences, bachelor’s degree) and high level (master’s degree or higher). For voting intentions, the answer options were: ‘the National Coalition Party’; ‘the Social Democrats’; ‘the Centre Party’; ‘the Green League’; ‘the Finns Party’; ‘the Left Alliance’; ‘other party’; ‘I don’t know’; ‘I am not going to vote’ and ‘I don’t want to say’. The last four options were combined into one category.

The variation in trust was tested using the chi-square test. Trust in THL and the ministry was examined using binary logistic regression, with gender, age, education and voting intentions as explanatory factors. The results are reported using odds ratios (ORs) and 95% confidence intervals. The models’ power was calculated using Nagelkerke’s pseudo R squared coefficient.

The study was conducted in accordance with the guidelines by the Finnish National Board on Research Integrity (TENK). An ethical review was not compulsory and was deemed unnecessary for confirming the study’s ethicality for the following reasons: the study does not fall under the Finnish Medical Research Act and Decree, and hence, Tampere University does not require an ethics board review. Given the complete anonymity, this study cannot lead to the identification or harm of any respondent. Finally, participants willingly engaged through Kantar Oy, a reputable company, ensuring their awareness of participation in research.

## Results

**Figure 2.**
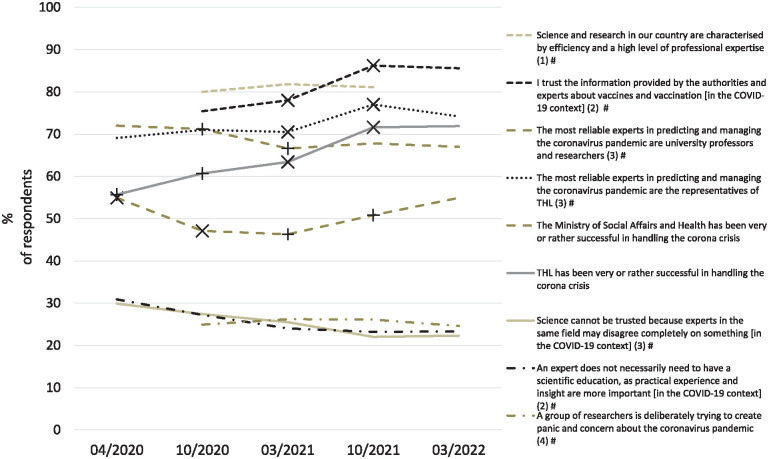
Trust in science, scientific institutions and the Ministry of Social Affairs and Health, in April 2020, October 2020, March 2021, October 2021 and March 2022 (*n*=5448).

Throughout the pandemic, a majority of respondents held a view that Finnish science is of high quality, that the experts of both THL and universities are the most trustworthy in predicting and managing the pandemic and trusted the vaccine information of the experts (Figure 2). Trust in the Ministry of Social Affairs and Health was lower. Statements reflecting lack of trust in science were supported by less than a fourth or a third of the respondents.

Looking at the variation throughout the study period, THL experts were increasingly perceived as the most reliable COVID-19 experts (*P*=0.007), THL was increasingly perceived as performing well (*P*<0.001) and the trust in vaccination information given by health authorities and experts grew (*P*<0.001). Contradictory views held by experts were observed less often as a reason to distrust science (*P*<0.001), and scientific education was increasingly seen as important for an expert to have (*P*<0.001). Only trust in university experts in COVID-19 issues declined (*P*=0.011). In addition, some changes may be pinpointed to have taken place between two measurements: opinion that THL has performed well increased between spring and autumn 2020. Between spring and autumn 2021, opinion that THL has performed well, trust in THL experts and trust in experts’ and authorities’ vaccine information increased. In the early-pandemic, trust in the ministry declined and recovered later.

The predictors of trust in THL and the ministry showed that in all models, women trusted these institutions more often than men did (Table III). In all models the age groups of 50 years and older trusted both THL and the ministry more than the youngest group did. Regarding the level of education, those with a middle or high level of education trusted THL more typically than those with only a basic education.

**Table III.** Opinion that the National Institite for Health and Welfare (THL) and the Ministry of Social Affairs had been very or or rather successful in handling the corona crisis, by background variables and voting intentions, unadjusted and adjusted odd ratios (*n*=5448).

	Trust in THL				Trust in the Ministry of Social Affairs and Health			
	Unadjusted		Adjusted		Unadjusted		Adjusted	
	OR	95% CI	OR	95% CI	OR	95% CI	OR	95% CI
Gender	***		***		***		***	
Men	1		1		1		1	
Women	1.408***	(1.259–1.574)	1.326***	(1.176–1.496)	1.512	(1.358–1.682)	1.434***	(1.279–1.609)
Age	***		***		*		***	
Under 30 years	1		1		1		1	
30–39 years	0.880	(0.722–1.073)	0.815	(0.660–1.007)	0.896	(0.736–1.091)	0.878	(0.711–1.083)
40–49 years	1.017	(0.832–1.244)	1.048	(0.844–1.302)	0.926	(0.759–1.129)	1.061	(0.856–1.315)
50–59 years	1.306**	(1.074–1.588)	1.292*	(1.047–1.594)	1.258*	(1.040–1.521)	1.410***	(1.148–1.732)
60–69 years	1.892***	(1.552–2.307)	1.693***	(1.371–2.091)	1.553***	(1.287–1.874)	1.520***	(1.242–1.859)
70 and more years	1.865***	(1.534–2.267)	1.679***	(1.363–2.068)	1.600***	(1.329–1.927)	1.670***	(1.367–2.040)
Education	***		**					
Basic level	1		1		1		1	
Middle level	1.377***	(1.215–1.561)	1.205**	(1.052–1.380)	1.102	(0.977–1.242)	1.012	(0.888–1.153)
High level	1.406***	(1.210–1.635)	1.240*	(1.053–1.461)	0.971	0.842–1.121)	0.909	(0.777–1.062)
Voting intentions	***		***		***		***	
Finns Party	1		1		1		1	
National Coalition Party	3.583***	(2.871–4.470)	2.819***	(2.234–3.557)	2.269***	(1.810–2.846)	1.897***	(1.497–2.402)
Centre Party	3.633***	(2.782–4.745)	2.965***	(2.251–3.907)	3.837***	(2.941–5.004)	3.276***	(2.494–4.303)
Green League	4.808***	(3.727–6.201)	4.793***	(3.673–6.255)	5.132***	(3.993–6.596)	4.888***	(3.770–6.338)
Social Democratic Party	5.459***	(4.281–6.961)	4.822***	(3.755–6.193)	7.150***	(5.598–9.133)	6.600***	(5.138–8.478)
Left Alliance	4.396***	(3.279–5.894)	3.952***	(2.923–5.345)	5.944***	(4.443–7.954)	5.456***	(4.051–7.348)
Other party, don't know etc.	2.250***	(1.874–2.703)	1.937***	(1.601–2.345)	2.293***	(1.881–2.795)	2.042***	(1.665–2.503)
Survey	***		***		***		***	
April 2020	1		1		1		1	
October 2020	1.230*	(1.037–1.458)	1.283**	(1.072–1.534)	0.732***	(0.619–0.866)	0.713***	(0.597–0.851)
March 2021	1.378***	(1.166–1.630)	1.435***	(1.204–1.712)	0.708***	(0.601–0.834)	0.682***	(0.573–0.811)
October 2021	1.999***	(1.673–2.389)	2.046***	(1.697–2.466)	0.848	(0.715–1.002)	0.809*	(0.677–0.967)
March 2022	2.031***	(1.701–2.426)	2.113***	(1.754–2.546)	1.001	(0.846–1.184)	1.002	(0.839–1.196)
			Nagelklerke 0.129				Nagelklerke 0.093	

CI: confidence interval; OR: odds ratio.

* *P*<0.05; **P<0.01; ***P<0.001.

In the context of voting intentions, in all models, supporters of the Finns Party demonstrated lower levels of trust in THL and the ministry than voters of all other political parties. In the adjusted models, the supporters of the Left Alliance, the Greens and the Social Democrats stood out as trusting THL and the ministry most typically.

Finally, with THL the opinion that it had performed well increased whereas for the ministry the trend was the opposite.

The Nagelkerke R^2^ shows that the models had only limited explanatory power.

## Discussion

Several survey items indicated a high level of trust in science, experts and scientific institutions throughout the first 2 years of the pandemic. Moreover, there was a rise in trust in THL and its experts in managing the pandemic, as well as in the confidence in the vaccination information provided by authorities and experts.

In contrast, trust in university researchers and professors in predicting and managing the pandemic and the opinion that the ministry performed well in managing the corona crisis declined. In the case of the ministry the situation recovered towards the end of the pandemic. The declining trust in university experts may be partly explained by the central role of THL in formulating pandemic policies, leading to highlighting trust in THL in contrast to trust in universities. The decline in trust in the ministry may be partly explained by the reluctance of the government to introduce mask recommendations in autumn 2020, and the contradictions between THL and the ministry. It is possible that the media portrayed THL as representing scientific facts, while the ministry was presented as exercising citizen control, often perceived negatively.

The increasing trust in THL and experts’ vaccination advice, especially around the mid-pandemic, along with the concurrent recovery of trust in the Ministry of Social Affairs and Health, suggests that beyond the possible initial rally effect [13], trust in science and authorities may also improve later.

Just before this increase in trust in the October 2021 survey, the number of inpatients and deaths increased [25, 27]. Later, at the time of the fifth survey, these numbers were at their height thus far. Furthermore, THL shifted its recommendations many times, notably on masks (between the spring and autumn of 2020) and the AstraZeneca vaccine (spring 2021). These types of changes in policy recommendations could deteriorate public trust in science [10]. This prompts consideration that trust in science and experts is influenced not solely by the trajectory of the pandemic, but also by a variety of other contributing factors.

First, many uncertainties of the early stages of the pandemic had changed to a better understanding of the course of the pandemic, more advanced treatment of the disease, and ongoing vaccination programme. Moreover, growing pandemic fatigue and indifference towards the virus may have led some individuals to relax their criticism of public health authorities.

Second, the high level of trust in science and scientific institutions prior to the pandemic [4, 5] may have helped many people interpret scientists’ changing estimations and recommendations as a normal part of science, rather than as signs of failure. Third, crisis management and media communication succeeded in reassuring the population of the legitimacy of public health authorities [29]. This was supported by the pandemic not being overtly politicised by major political parties.

In agreement with earlier research [22, 23], age and education predicted trust in THL and the ministry. The intention to vote for the Finns Party was a significant indicator of distrust in THL, aligning with previous reports that Finns Party supporters are often more sceptical of science [4]. This is similar to the US, where value conservatism has been linked to science distrust in the COVID-19 context [22]. Our study showed a similar association with the ministry responsible for protecting public health.

Much like many of its European counterparts [14], the Finns Party largely avoided overtly politicising the pandemic and responses to it. Nevertheless, the party’s supporters expressed a more sceptical stance towards THL, suggesting that this inclination runs deeper in the thinking of the party and its supporters [30]. We suggest that those who did not trust public health authorities had found their home among the Finns Party already before the pandemic – a party with a history of science scepticism. At the same time populist COVID-19 rhetoric in the public sphere resonated with those who were already leading towards the Finns Party.

Regarding the limitations of the study, those with basic education were somewhat underrepresented. However, we did not find big differences across education levels, suggesting that a stronger representation of elementary-educated individuals may not significantly alter our results. Moreover, we did not follow the same individuals over time, so some changes might result from different types of participants. For example, more sceptical people might have been less willing to join as the pandemic progressed.

## Conclusions

Trust in major public health institutions is essential for ensuring public compliance with public health efforts [29]. The study proved that Finns maintained a high level of trust in major scientific institutions throughout the pandemic. Young, men and supporters of the right-wing Finns Party exhibited a lower level of trust in the THL and the ministry. However, not even the supporters of the Finns Party should simply be labelled as an anti-science, because in them, there are many who were not among the critics.

However, given the strong association between the voting intention and criticism of THL and the ministry, future public health initiatives should consider tailoring their messages on social media and news media to resonate better with all population groups. In increasingly politically polarised societies, political sentiments should be analysed in a public health crisis of varying severity and extent.

## References

[1] Palonen E. Finland: Political developments and data in 2021: local elections and negotiating the post-pandemic opening. Eur J Polit Res Polit Data Yearbook 2022;61:147–159.

[2] PytlikZillig LM Kimbrough CD. Consensus on conceptualizations and definitions of trust: are we there yet? Cham: Springer, 2016.

[3] Cairney P Wellstead A. COVID-19: effective policymaking depends on trust in experts, politicians, and the public. Policy Design Pract 2021;4:1–14.

[4] Saarinen A Koivula A Keipi T. Political trust, political party preference and trust in knowledge-based institutions. Int J Sociol Soc Policy 2020;40:154–168.

[5] The Finnish Society for Scientific Information. Science Barometer 2019. Helsinki: The Finnish Society for Scientific Information, 2019.

[6] Uscinski J Enders A Klofstad C , et al. Why people believe in COVID-19 conspiracy theories? Harvard Kennedy School Misinform Rev 2020;1:1–12.

[7] Vieten UM. The “new normal” and “pandemic populism”: The COVID-19 crisis and anti-hygienic mobilisation of the far-right. Soc Sci 2020;9:1–14.

[8] Pennycook G McPhetres J Bago B , et al. Beliefs about COVID-19 in Canada, the United Kingdom, and the United States: a novel test of political polarization and motivated reasoning. Personal Soc Psychol Bull 2022;48:750–765. 10.1177/01461672211023652 PMC9066691 34180276

[9] Zapp M. The legitimacy of science and the populist backlash: cross-national and longitudinal trends and determinants of attitudes toward science. Public Understand Sci 2022;31:885–902. 10.1177/09636625221093897 35713397

[10] Kreps SE Kriner DL. Model uncertainty, political contestation, and public trust in science: evidence from the COVID-19 pandemic. Sci Adv 2020;6:eabd4563. 10.1126/sciadv.abd4563 PMC7577608 32978142

[11] Gustafson A Rice RE. A review of the effects of uncertainty in public science communication. Public Understand Sci 2020;29:614–633. 10.1177/0963662520942122 32677865

[12] Jørgensen F Bor A Rasmussen MS , et al. Pandemic fatigue fueled political discontent during the COVID-19 pandemic. Proc Natl Acad Sci 2022;119:e2201266119. 10.1073/pnas.2201266119 PMC9860270 36413499

[13] Esaiasson P Sohlberg J Ghersetti M , et al. How the coronavirus crisis affects citizen trust in institutions and in unknown others: evidence from ‘the Swedish experiment’. Eur J Polit Res 2021;60:748–760.

[14] Bobba G Hubé N. Populism and the Politicization of the COVID-19 Crisis in Europe. Cham: Palgrave MacMillan/Springer Nature, 2021.

[15] Palamenghi L Barello S Boccia S , et al. Mistrust in biomedical research and vaccine hesitancy: the forefront challenge in the battle against COVID-19 in Italy. Eur J Epidemiol 2020;35:785–788. 32808095 10.1007/s10654-020-00675-8 PMC7431109

[16] Agley J. Assessing changes in US public trust in science amid the COVID-19 pandemic. Public Health 2020;183:122–125. 32405095 10.1016/j.puhe.2020.05.004 PMC7218345

[17] Algan Y Cohen D Davoine E , et al. Trust in scientists in times of pandemic: panel evidence from 12 countries. Proc Natl Acad Sci 2021;118:e2108576118. 10.1073/pnas.2108576118 PMC8501808 34580225

[18] Luna DS Bering JM Halberstadt JB. Public faith in science in the United States through the early months of the COVID-19 pandemic. Public Health Pract 2021;2:100103. 10.1016/j.puhip.2021.100103 PMC8555978 34746892

[19] Groeniger JO Noordzij K Van Der Waal J , et al. Dutch COVID-19 lockdown measures increased trust in government and trust in science: a difference-in-differences analysis. Soc Sci Med 2021;275:113819. 33725488 10.1016/j.socscimed.2021.113819 PMC9756755

[20] Bromme R Mede NG Thomm E , et al. An anchor in troubled times: trust in science before and within the COVID-19 pandemic. PloS One 2022;17:e0262823. 10.1371/journal.pone.0262823 PMC8827432 35139103

[21] Battiston P Kashyap R Rotondi V. Reliance on scientists and experts during an epidemic: evidence from the COVID-19 outbreak in Italy. SSM – Popul Health 2021;13:100721. 33553567 10.1016/j.ssmph.2020.100721 PMC7859315

[22] Latkin CA Dayton L Moran M , et al. Behavioral and psychosocial factors associated with COVID-19 skepticism in the United States. Curr Psychol 2022;41:7918–7926. 33424206 10.1007/s12144-020-01211-3 PMC7786141

[23] Jallinoja P Väliverronen E. Suomalaisten luottamus instituutioihin ja asiantuntijoihin COVID19-pandemiassa. Media & Viestintä 2021;44:1–24.

[24] Scheitle CP Corcoran KE. COVID-19 skepticism in relation to other forms of science skepticism. Socius 2021;7:1–12.

[25] Tiirinki H Sovala M Jormanainen V , et al. COVID-19 endemic phase in Finland: an analysis of health policies and vaccination strategy. Health Policy Technol 2024;13:100800.

[26] Palonen E. Finland: Political Developments and Data in 2020. Eur J Polit Res Polit Data Yearbook 2021;60:132–140.

[27] Finnish Institute for Health and Welfare. Situation update on coronavirus. 2024. https://thl.fi/en/topics/infectious-diseases-and-vaccinations/what-s-new/coronavirus-covid-19-latest-updates/situation-update-on-coronavirus (2024, accessed 22 August 2024).

[28] Statistics Finland. Population structure and educational structure of population. 2022. https://stat.fi/en/statistical-data (2022; accessed 13 October 2024).

[29] Lund-Tønnesen J Christensen T. The dynamics of governance capacity and legitimacy: the case of a digital tracing technology during the COVID-19 pandemic. Int Public Manage J 2023;26:126–144.

[30] Ylä-Anttila T. Populist knowledge: ‘Post-truth’ repertoires of contesting epistemic authorities. Eur J Cult Polit Sociol 2018;5:356–388.

